# Front-of-Package-Label-Style Health Logos on Menus—Do Canadian Consumers Really Care about Menu Health Logos?

**DOI:** 10.3390/nu16040545

**Published:** 2024-02-16

**Authors:** Yahan Yang, Sylvain Charlebois, Janet Music

**Affiliations:** 1 Department of Nutritional Sciences, Temerty Faculty of Medicine, University of Toronto, Toronto, ON M5S 1A8, Canada; 2 Agri-Food Analytics Lab, Dalhousie University, Halifax, NS B3H 4R2, Canada; sylvain.charlebois@dal.ca; 3 Faculty of Arts & Social Sciences, Dalhousie University, Kenneth C. Rowe Management Building, Halifax, NS B3H 4R2, Canada

**Keywords:** nutritional policy, restaurant foods, consumer research, menu labelling

## Abstract

Public health policies have been widely utilized to improve population nutrition, such as the newly announced front-of-pack labels (FOPLs) that will be applied to Canadian prepackaged foods to help consumers make healthier selections. However, research on similar health logos in the food service sector has been limited. This study explores the potential application of FOPL-style health logos in the food service sector and its impact on consumer behaviors. A survey was conducted among 1070 Canadians to assess their awareness, perception, and support for health logos on restaurant menus. The results indicate that while participants value healthy food options when dining out, taste, price, and convenience remain the primary factors influencing their choices. Most participants were unaware of existing FOPL policies and demonstrated mixed responses regarding the influence of similar health logos on their restaurant selection. However, a majority expressed a desire to see FOPL-style health logos on menus, and nutrient profile ratings and logos indicating nutrient limitations or encouragements were listed as preferred health logos. Notably, females indicated higher supportiveness for FOPL-style health logos on menus and individuals with food allergies exhibited higher agreement in the likelihood of eating at a restaurant displaying labels. Additionally, findings revealed that FOPL-style health logos alone may not significantly deter consumers from purchasing labelled menu items, especially if price is affected. Overall, this study highlights the need for further understanding consumer perceptions to effectively develop and implement FOPL initiatives in the food service sector.

## 1. Introduction

Front-of-package labelling

Dietary risks, as determined by 15 dietary diet quality components (e.g., high in sodium, low in vegetables), were accountable for 8 million global attributable deaths in 2019, according to the Global Burden of Disease Study [1]. To improve population nutrition, many policy frameworks and guidelines have been applied in public health, including individual, organizational, and system-level interventions [2,3]. One of these strategies iss front-of-package labelling (FOPL), which uses symbols that summarize important nutritional characteristics of foods on the display surface of packages, such as a warning label that highlights nutrients which should be limited in the diet [4,5]. The utilization of FOPL has demonstrated efficacy in improving the overall nutritional quality of a population by enhancing consumer nutrition knowledge, facilitating healthier food choices, and encouraging food reformulations [6,7,8,9,10]. Three systematic reviews showed that FOPL has a positive effect on consumer purchasing, such as a lower sugar and sodium content of the purchased foods, although evidence on consumption was limited [7,8,9]. Song et al. specifically compared different types of labels (e.g., traffic light labels, nutrient warnings) and showed that warning labels are associated with better overall healthfulness and reduced energy in purchases [9]. Similarly, Ikonen et al. found that although different types of FOPL all have significantly positive impacts on consumers choosing healthier products, warning labels showed the strongest impact [7]. As of 2023, 16 countries had introduced mandatory FOPLs, and 10 of them introduced warning labels [10]. In June 2022, Canada introduced its mandatory FOPL regulations for nutrients of concern, including saturated fat, sodium, and sugar, with implementation slated for completion by 2026 [6].

Labelling in the food service sector

While FOPL has been widely studied on prepackaged foods, research and policies related to labelling in the food services sector have been much scarcer. Menu labelling policies usually focus on displaying the energy content, as several countries (e.g., United States, United Kingdom) have national-level mandatory calorie labelling in large chain restaurants [11]. Several systemic reviews that examined the effects of calorie labelling on foods purchased or consumed in real-world and laboratory settings showed mixed results, concluding that impacts are limited [12,13,14]. There has been no national policy on health logos on menus worldwide, and New York City and Philadelphia were the first two cities to put sodium warning icons next to restaurant menu items that contain more than 2300 mg of sodium [15].

In Canada, while there are mandatory labelling regulations on prepackaged foods [16], there is currently no federal labelling regulation specific to the food service sector, except for a mandate in Ontario that requires food service establishments with 20 or more outlets to display energy content information on their menus [17]. Considering the significant proportion of Canadians who consume meals at restaurants [18,19] and the poor nutritional quality of Canadian restaurant foods [20], strategies to improve diet quality in the food service sectors become necessary.

Given the significance of understanding consumer perceptions in developing and improving nutrition policy initiatives, this study aims to explore the potential application of comprehensive nutritional information in the food service sector, focusing on three primary research questions: (1) the desire of Canadian consumers to have increased nutritional information presented by health logos on menus and within the food service sector, like the FOPLs designed for prepackaged foods, (2) the demographic groups most likely to be affected by such information, and (3) the potential impact on consumer behaviors within the restaurant sector resulting from enhanced access to nutritional information presented by these health logos.

## 2. Materials and Methods

This study employed a cross-sectional survey design to assess the potential application of FOPL in the food service sector. A voluntary survey in both English and French was administered to participants across Canada using the online survey platform Qualtrics, in collaboration with Angus Reid, a well-established Canadian field house and a member of the Canadian Marketing Association.

Respondents were recruited through a broad invitation method and a double opt-in screening process for recruitment. Potential participants were engaged through targeted banner advertisements on various websites and partnerships with non-governmental and charitable organizations. This strategy ensures a representative demographic mix that encompasses diversity across all population subgroups. Ethics approval was granted by the Institutional Review Board of Dalhousie University REB No. 2023-6677 (26 May 2023). Participants were required to have lived in Canada for at least 12 months and to be at least 18 years of age. Participants needed to provide informed consent by clicking on a link at the end of the consent form if they agreed to proceed with completing the survey, and the consent could be rescinded by closing their browser.

Data were collected between 1 June and 30 June 2023, and a total of 1070 participants were recruited for the study. Results will show varying totals due to incompletes. Participants were selected from the general population of Canada and were diverse in terms of age, gender, marital status, education level, household income, and dietary restrictions. The demographic characteristics of the participants are summarized in Table 1.

Of the 1070 participants, more than 50% were Millennials (born 1981–1996) and Baby Boomers (born 1946–1964). Of the participants, 50% were female and 49% were male, and 61% of were married. Most participants’ education levels were university (37%) and community college, technical college, or CEGEP (24%), followed by post-graduate degree (17%). Sixty-seven percent of participants reported having no dietary restrictions and 25% had food allergies and/or intolerances.

The survey consisted of questions related to participants’ perceptions and supportiveness towards health logos on restaurant menus, their restaurant behaviors, and their attitudes towards FOPL-style health logos. The survey included closed-ended questions, rating scales, and multiple-choice questions. Participants were asked whether they had seen health logos on restaurant menus and their perceptions towards them. The types of health logos and their prevalence were assessed. Participants’ agreement or disagreement with statements related to the influence of health logos on their restaurant choices was measured.

**Figure 1:**
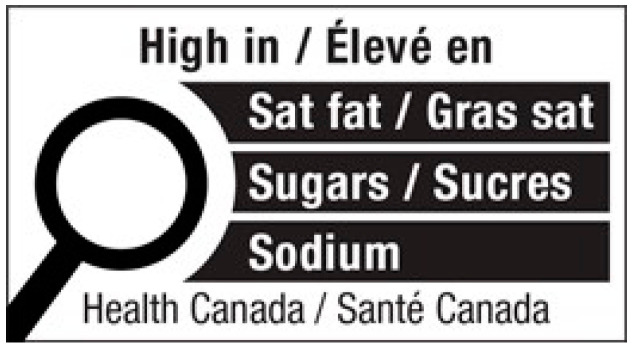
Front-of-pack labelling that will be applied to prepackaged foods.

**Figure 2:**
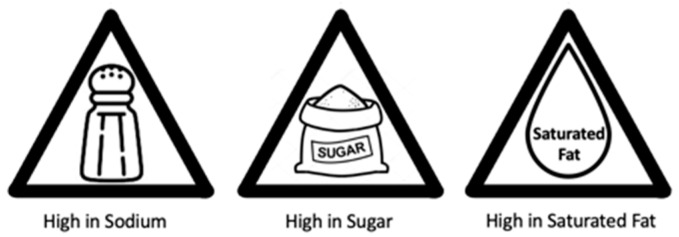
Proposed design of a FOPL-style health logo for ‘high in sodium’, ‘high in sugar’, and ‘high in saturated fat’.

Participants were asked about their frequency of visiting restaurants, the proportion of their diet composed of foods prepared away from home, and their preferences for different types of restaurants. Participants were presented with the FOPL published by Health Canada (Figure 1) and asked about their awareness and support for applying this FOPL on menus. Participants were also shown triangle warning sign designs and asked about their perceived effectiveness compared to FOPLs for prepackaged foods (Figure 2). Participants’ preferences for different types of health logos on menus were assessed. Participants’ likelihood of eating at a restaurant with labels displayed on the menu and their willingness to purchase menu items with warning labels were measured.

Descriptive statistics were used to summarize the demographic characteristics of the participants and the survey responses. Frequencies and percentages were reported for categorical variables. Ordinal logistic regression was used to analyze independent variables including age, gender, marital status, income, education levels, dietary restrictions, and frequency of eating out. Statistical significance was set at *p* < 0.05.

## 3. Results

### 3.1. Eating out Behaviors of Participants

**Figure 3:**
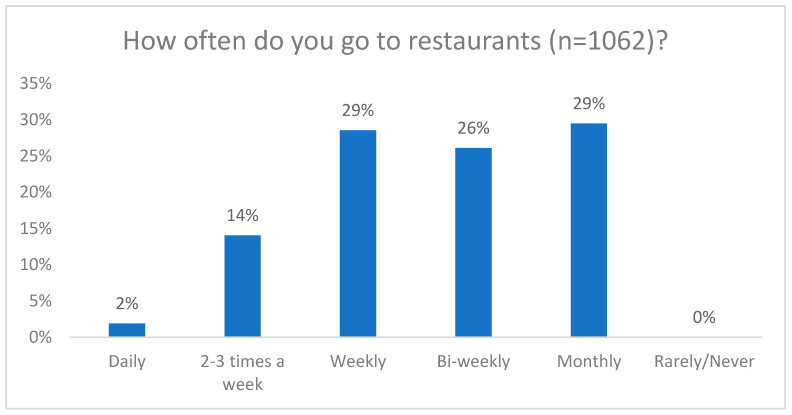
How often do you go to restaurants?

The results of this study indicate that participants displayed a regular frequency of visiting restaurants, with all participants reporting at least monthly visits and 45% visiting restaurants on a weekly basis (Figure 3).

**Figure 4:**
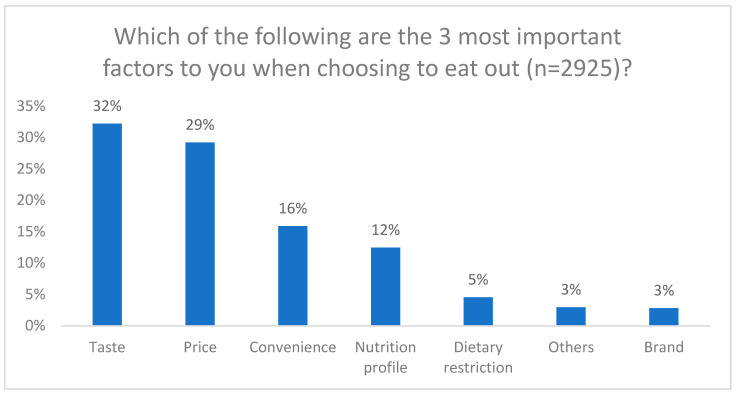
Which of the following are the 3 most important factors to you when choosing to eat out?

However, most participants (81%) stated that food prepared away from home accounted for only 0–25% of their overall diet, and a mere 4% reported that over half of their diet consisted of food prepared away from home. Among the various types of restaurants, sit-down establishments were the most visited (41%), followed by fast-food restaurants (28%), coffee shops (20%), and bakery/dessert shops (9%). When making choices about dining out, participants prioritized factors such as taste (32%), price (29%), and convenience (16%), with the nutrition profile ranking fourth in importance (12%) (Figure 4).

Nevertheless, an average of 80% of participants agreed with the notion that having healthy food options when dining out is important. This percentage was significantly higher (84%) among those who attended university or post-graduate degrees compared to those who did not graduate from high school (69%) (Supplementary Table S1, *p* < 0.05). Significantly more female participants (84%) than male participants (75%) agreed with the statement (*p* < 0.001). Baby Boomers (80%) and Millennials (81%) had a higher percentage of agreement compared to the Silent Generation (63%) (*p* < 0.05). Interestingly, people with food allergy/intolerance and faith-based dietary restrictions (53%) had a higher percentage of agreement compared with those without dietary restrictions (79%) (*p* < 0.05). These findings shed light on the participants’ restaurant behaviors and preferences, suggesting that while taste, price, and convenience are primary considerations, there is a prevailing acknowledgment of the significance of healthy food options in the dining out context.

### 3.2. Perception of Menu Health Logos

**Figure 5:**
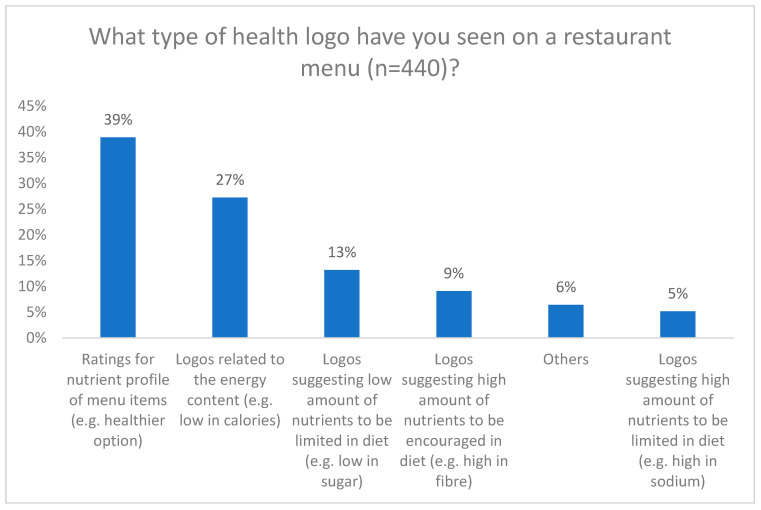
What type of health logo have you seen on a restaurant menu?

Among the respondents, 43% reported observing a health logo on restaurant menus. Within this subset, the most prevalent types of logos were those indicating ratings for the nutrient profile of menu items and logos related to energy content (Figure 5). Conversely, warning labels were the least commonly encountered. Additional responses included logos indicating gluten-related claims and vegan/vegetarian claims. When queried about whether the presence of health logos influences their choice of restaurants, respondents displayed mixed opinions. Approximately 39% of participants neither agreed nor disagreed, while similar percentages of participants indicated disagreement (32%) and agreement (30%). A higher percentage of female participants (33%) and married participants (31%) selected ‘agree’ compared to male participants (25%) and single participants (23%) (*p* = 0.01 and 0.02, respectively) (Supplementary Table S1). Merely 20% of participants agreed that the individuals they dine with influence their decisions regarding dishes with or without health logos, with the majority either disagreeing (50%) or expressing neutrality (39%). However, a higher percentage of participants who eat out 2–3 times a week (29%) and daily (40%) stated they ‘agree’ with this statement (*p* = 0.02 and *p* = 0.03, respectively).

When participants were presented with the FOPL logo published by Health Canada, it was found that a significant proportion of participants, specifically 84%, were unaware of this policy. However, 59% of participants expressed their desire to see a similar FOPL-style health logo on menus. When stratifying by gender, 10% more female respondents compared to male respondents selected ‘somewhat agree’ or ‘strongly agree’ (*p* = 0.01) (Supplementary Table S1). Moreover, when participants were shown the triangle warning sign design (Figure 2), a considerable 65% agreed that it would be more effective than the current FOPL used for prepackaged foods (Supplementary Table S1).

**Figure 6:**
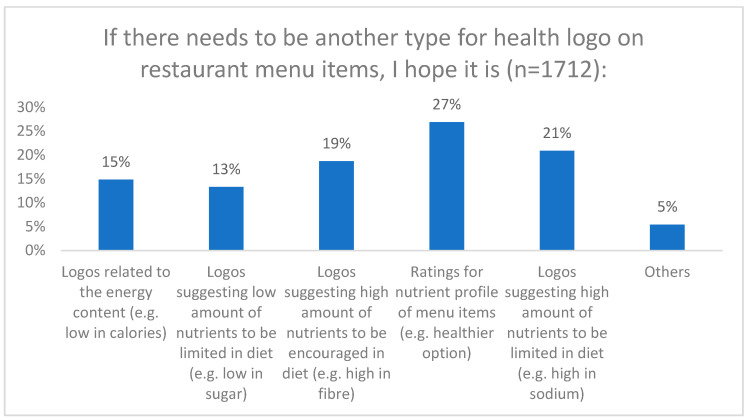
If there needs to be another type for health logo on restaurant menu items, I hope it is.

Regarding the types of health logos participants expressed interest in seeing on menus, the highest responses were observed for ratings indicating the nutrient profile of menu items (27%), followed by logos suggesting a high amount of nutrients to be limited in the diet (21%), and logos suggesting a high amount of nutrients to be encouraged in the diet (19%) (Figure 6).

**Figure 7:**
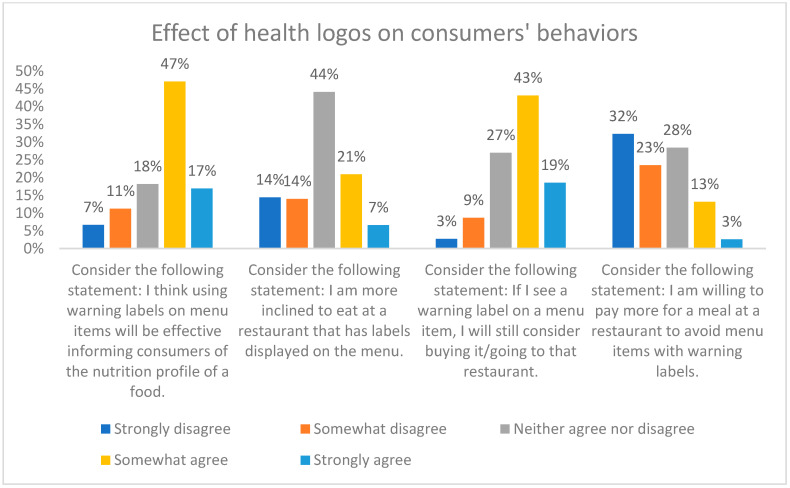
Effect of health logos on consumers’ behaviors.

Despite the majority (64%) of participants agreeing that the use of warning labels on menu items would effectively inform consumers about the nutritional profile of food (Figure 7), questions exploring the potential impact of menu labelling on consumer behaviors revealed contrasting responses. Overall, only 28% of participants agreed that they were more likely to eat at a restaurant displaying labels on the menu. Also, 62% agreed that they would still consider purchasing a menu item or visiting a restaurant even if a warning label was present. When stratified by education level, respondents that did not attend high school had the highest percentage of selecting ‘disagree’ and the proportion was significantly different from other groups (*p* < 0.05) (Supplementary Table S1). Additionally, a mere 16% of participants agreed that they were willing to pay a higher price for a meal at a restaurant to avoid menu items with warning labels, whereas 32% and 23% of participants expressed strong or moderate disagreement, respectively (Figure 7). However, among participants who eat out daily, 35% agreed that they would pay a higher price, significantly higher than the participants who eat out monthly (*p* = 0.02).

## 4. Discussion

The findings of this study provide insights into restaurant behaviors and preferences among the participants, shedding light on their dining out habits and the significance of healthy food options. They contribute to the understanding of consumer attitudes towards FOPL and inform the development of initiatives aimed at improving population health through FOPL-style health logos in the food service sector.

Although the eating out frequency reported in this study (45%) was lower than that reported pre-pandemic in 2019 (54%) [18], restaurants still have a role in the participants’ overall food consumption patterns, even after the pandemic. However, despite the frequency of restaurant visits, the participants’ reliance on food prepared away from home is relatively low, with most people reporting that it accounted for a small proportion of their overall diet. This finding suggests that while dining out is a common practice, it does not dominate their daily food intake. Additionally, only a 4% proportion of participants reported that over half of their diet consisted of food prepared away from home. These results highlight the importance of considering home-cooked meals and other sources of nutrition when evaluating the overall dietary habits of individuals. A multi-faceted approach such as the Health Eating Strategy [21] will be necessary to improve the dietary habits of Canadians at the individual, industrial, and governmental levels.

When analyzing the types of restaurants participants frequented, sit-down establishments emerged as the most visited, followed by fast-food restaurants, coffee shops, and bakery/dessert shops. Previous research has shown that less healthful eating behaviors are mainly associated with eating out at fast food restaurants, rather than at sit-down restaurants [22,23]. Understanding these preferences is crucial for restaurant owners and policymakers to tailor their offerings and interventions accordingly.

Regarding the factors influencing participants’ choices when dining out, taste, price, and convenience were identified as the primary considerations. Notably, nutrition profiles ranked fourth in importance. These findings align with previous research, highlighting that taste and cost often outweigh health considerations when making food choices outside the home [24]. However, it is noteworthy that despite the lower ranking of nutrition in the choice factors, the majority of participants expressed agreement with the importance of having healthy food options when dining out. This demonstrates a general awareness and acknowledgement among participants that healthful choices are essential when eating away from home.

This study also explored the awareness and influence of health logos on restaurant menus. While 43% of participants reported observing health logos, ratings for nutrient profiles and energy content were the most prevalent types. Warning labels were less commonly encountered, indicating a potential area for improvement in menu labelling initiatives. Participants’ opinions on the influence of health logos on their restaurant choices were mixed, with a notable percentage neither agreeing nor disagreeing. Also, although most participants were unaware of Health Canada’s FOPL policy, a considerable percentage expressed a desire to see a similar FOPL-style health logo on restaurant menus. This highlights the potential for menu labelling initiatives to increase consumer awareness of nutritional information and promote healthier choices, but also suggests that the impact of health logos on consumer behavior requires further investigation and possibly a design specifically tailored to menus will be required. This study’s results highlighted participants’ interest in specific types of health logos on menus, with a focus on nutrient profile ratings and logos suggesting nutrients to be limited or encouraged in the diet. A quasi-experimental trial in France implemented the Nutri-Score system, a summary graded coloured system, in catering and found a significant reduction in the intake of calories, sugars, and saturated fats among participants [25]. Similarly, an Australian study demonstrated the feasibility of applying the Health Star Rating system to restaurant foods [26]. These studies and results provide valuable insights for policymakers and restaurant owners in designing effective menu labelling systems that align with consumers’ interests and needs.

Interestingly, the significance of healthy food options and perceptions of health logos varied based on respondents’ age, gender, education level, and dietary restrictions. For example, participants with higher education levels (university or post-graduate degree) were more likely to emphasize the importance of healthy food options, indicating a positive association between education and health-conscious choices, which was shown in many previous studies [27,28]. Additionally, female participants displayed a higher agreement with the importance of healthy food options and were more likely to support the implementation of FOPL-style health logos than males, suggesting potential gender differences in health-related preferences in the restaurant setting, echoing those found in previous nutrition behavior research [29]. These differences underscore the importance of targeted interventions and tailored communication strategies for different demographic groups.

Moreover, the potential impact of menu labelling on consumer behavior is complex. While the majority of participants agreed that warning labels would effectively inform consumers about the nutritional profile of food, most participants expressed neutrality in whether they were likely to eat at a restaurant displaying labels on the menu. This suggests that menu labelling alone might not be sufficient to drive significant changes in dining out habits. The U.K. has published sodium guidelines for the eating out, takeaway, and delivery sectors by 2024 [30]. Canada has its sodium target for packaged foods but have not yet extended to the food service sector [31]. Therefore, thorough nutrition guidelines and regulations targeting the restaurant foods will also be necessary so that healthier options are available for consumers.

Furthermore, the willingness to pay a higher price for a meal to avoid menu items with warning labels was very low, indicating that price remains a significant factor in dining out decisions, even in the presence of health-related information. This could be concerning as poor diet quality is particularly evident among Canadians with a lower socioeconomic status [32]. Even if warning labels are presented on unhealthy menu items, lower-income consumers may still purchase them if healthier options are more expensive. Therefore, equity-oriented policies that improve economic security would be more beneficial than information-based nutrition policies in these scenarios.

Overall, these results provide valuable insights into restaurant behaviors and preferences, emphasizing the need for a comprehensive approach to promote healthier dining out choices. While menu labelling initiatives hold promise, they should be complemented with other strategies, such as public awareness campaigns, menu reformulation, and collaborations with the food industry, to achieve meaningful and sustained changes in consumer behavior. Understanding the varying preferences and motivations across demographic groups will be crucial in developing effective and targeted interventions. Policymakers and restaurant owners can utilize these findings to better align their offerings with consumer needs and contribute to a healthier dining out environment. Future research can delve deeper into understanding the barriers to and facilitators of healthy dining out choices and evaluate the long-term impact of menu labelling initiatives on consumer behavior and public health outcomes.

This study has some limitations. Firstly, the sample was drawn from the general population of Canada and was limited to adults who were able to answer questionnaires in English or French, which may limit the generalizability of the findings to children/adolescents and to minority groups who do not communicate in English/French. Secondly, dietary behaviors were collected retrospectively, and self-reported data are subject to recall and response biases. This study examined consumer perceptions and may not represent actual behavioral changes in food purchases and consumptions associated with menu labels.

## 5. Conclusions

This study assessed the potential application of FOPL-style health logos in the food service sector and the data collected from the survey questionnaire provide insights into participants’ perceptions, preferences, and behaviors related to health logos on restaurant menus. Although participants do think it is important to have healthy food options available when dining out, a much lower number of participants think health logos will affect their choices of restaurants/foods. The two main reasons could be that 1. food away from home is not the major component of their diet and 2. taste, price, and convenience are the most important factors when dining out. This study highlights that FOPL-style health logos in the food services sector may not be sufficient and that more multidisciplinary strategies will be necessary to improve the dietary intake of Canadians.

## Figures and Tables

**Table 1:** Demographics of the study participants (n = 1070).

Age Group	Number	%
The Silent Generation: born 1928–1945	28	3%
Baby Boomers: born 1946–1964	341	32%
Generation X: born 1965–1980	238	22%
Millennials: born 1981–1996	393	37%
Generation Z: born 1997–2012	65	6%
Grand Total	1065	100%
Gender		
Female	536	50.3%
Male	522	49.0%
Other	3	0.3%
Prefer not to answer	4	0.4%
Grand Total	1065	100%
Marital Status		
Married	644	61%
Separated/Divorced	79	7%
Single	290	27%
Widowed	24	2%
Prefer not to answer	27	3%
Grand Total	1064	100%
Education Level		
Did not graduate high school	16	2%
High school graduate certificate or equivalent	137	13%
Community college, technical college, or CEGEP	253	24%
Trades certificate or diploma	87	8%
University (undergraduate degree)	392	37%
Post-graduate degree	180	17%
Grand Total	1065	100%
Household Income		
Under CAD 25,000	53	5%
CAD 25,000 to CAD 49,999	122	11%
CAD 50,000 to CAD 74,999	160	15%
CAD 75,000 to CAD 99,999	146	14%
CAD 100,000 to CAD 124,000	138	13%
CAD 125,000 to CAD 149,999	125	12%
Over CAD 150,000	190	18%
Prefer not to answer	132	12%
Grand Total	1066	100%
Dietary Restrictions		
No dietary restriction	718	67%
Allergies and/or intolerances, and faith-based restrictions (e.g., halal, kosher, etc.)	16	2%
Faith-based restrictions (e.g., halal, kosher, etc.)	18	2%
Food allergies and/or intolerances	271	25%
Others	42	4%
Grand Total	1065	100%

## Data Availability

The data presented in this study are available on request from the corresponding author.

## Supplementary Materials

The following supporting information can be downloaded at: https://www.mdpi.com/article/10.3390/nu16040545/s1, Supplementary Table S1. Participant responses stratified by age group, gender, marital status, education, income, dietary restrictions and eating out frequency.
