# Supplementary material for: Front-of-Package-Label-Style Health Logos on Menus—Do Canadian Consumers Really Care about Menu Health Logos?
Source: Nutrients. 2024 Feb 16;16(4):545. doi: 10.3390/nu16040545 (PMC10892900; doi:10.3390/nu16040545)
Supplement: Supplementary file 1 [file nutrients-16-00545-s001.zip › nutrients-2863053-supplementary.pdf]

# Supplementary Table

Supplementary Table S1. Participant responses stratified by age group, gender, marital status, education, income, dietary restrictions and eating out frequency.

| Group | Variable | Strongly disagree | Somewhat disagree | Neither agree nor disagree | Somewhat agree | Strongly agree | Total | p value |
|---|---|---|---|---|---|---|---|---|
| **Consider the following statement: I think it is important to have healthy food options when dining out.** | | | | | | | | |
| Age Group | The Silent Generation: Born 1928-1945 | 2 (7.4%) | 1 (3.7%) | 7 (25.9%) | 9 (33.3%) | 8 (29.6%) | 27 (2.5%) | |
| | Baby Boomers: Born 1946-1964 | 20 (5.9%) | 13 (3.8%) | 35 (10.3%) | 96 (28.2%) | 177 (51.9%) | 341 (32.2%) | 0.02* |
| | Generation X: Born 1965-1980 | 16 (6.8%) | 10 (4.2%) | 25 (10.6%) | 81 (34.3%) | 104 (44.1%) | 236 (22.3%) | 0.08 |
| | Generation Z: Born 1997-2012 | 1 (1.6%) | 6 (9.4%) | 9 (14.1%) | 25 (39.1%) | 23 (35.9%) | 64 (6.0%) | 0.10 |
| | Millennials: Born 1981-1996 | 11 (2.8%) | 15 (3.8%) | 48 (12.3%) | 138 (35.3%) | 179 (45.8%) | 391 (36.9%) | 0.04* |
| | Prefer not to answer | 0 (0%) | 0 (0%) | 0 (0%) | 1 (100.0%) | 0 (0%) | 1 (0.1%) | 0.64 |
| Gender | Male | 24 (4.6%) | 25 (4.8%) | 82 (15.8%) | 197 (37.9%) | 192 (36.9%) | 520 (49.1%) | p<0.001 *** |
| | Female | 25 (4.7%) | 20 (3.8%) | 42 (7.9%) | 152 (28.6%) | 293 (55.1%) | 532 (50.2%) | |
| | Other | 0 (0%) | 0 (0%) | 0 (0%) | 0 (0%) | 3 (100.0%) | 3 (0.3%) | |
| | Prefer not to answer | 1 (25.0%) | 0 (0%) | 0 (0%) | 0 (0%) | 3 (75.0%) | 4 (0.4%) | 0.36 |
| Marital Status | Single | 15 (5.2%) | 15 (5.2%) | 41 (14.3%) | 94 (32.9%) | 121 (42.3%) | 286 (27.0%) | |
| | Married | 27 (4.2%) | 27 (4.2%) | 73 (11.4%) | 209 (32.5%) | 307 (47.7%) | 643 (60.8%) | 0.05 |
| | Prefer not to answer | 2 (7.4%) | 0 (0%) | 2 (7.4%) | 7 (25.9%) | 16 (59.3%) | 27 (2.6%) | 0.10 |
| | Separated / Divorced | 6 (7.6%) | 2 (2.5%) | 6 (7.6%) | 30 (38.0%) | 35 (44.3%) | 79 (7.5%) | 0.49 |
| | Widowed | 0 (0%) | 1 (4.3%) | 2 (8.7%) | 9 (39.1%) | 11 (47.8%) | 23 (2.2%) | 0.22 |

| | | | | | | | | |
|---|---|---|---|---|---|---|---|---|
| Education | Did not graduate high school | 4 (25.0%) | 0 (0%) | 1 (6.2%) | 5 (31.2%) | 6 (37.5%) | 16 (1.5%) | |
| | Community college, technical college, or CEGEP | 12 (4.7%) | 11 (4.3%) | 36 (14.2%) | 80 (31.6%) | 114 (45.1%) | 253 (23.9%) | 0.19 |
| | High school graduate certificate or equivalent | 9 (6.6%) | 10 (7.3%) | 22 (16.1%) | 46 (33.6%) | 50 (36.5%) | 137 (12.9%) | 0.57 |
| | Post-graduate degree | 9 (5.0%) | 5 (2.8%) | 14 (7.8%) | 53 (29.6%) | 98 (54.7%) | 179 (16.9%) | 0.04* |
| | Trades certificate or diploma | 2 (2.4%) | 6 (7.1%) | 16 (18.8%) | 38 (44.7%) | 23 (27.1%) | 85 (8.0%) | 0.65 |
| | University (undergraduate degree) | 14 (3.6%) | 13 (3.3%) | 35 (9.0%) | 127 (32.6%) | 200 (51.4%) | 389 (36.7%) | 0.04* |
| Income | Under $25,000 | 3 (5.7%) | 3 (5.7%) | 10 (18.9%) | 12 (22.6%) | 25 (47.2%) | 53 (5.0%) | |
| | $100,000 to $124,000 | 8 (5.8%) | 8 (5.8%) | 14 (10.1%) | 44 (31.9%) | 64 (46.4%) | 138 (13.0%) | 0.71 |
| | $125,000 to $149,999 | 7 (5.6%) | 6 (4.8%) | 10 (8.1%) | 41 (33.1%) | 60 (48.4%) | 124 (11.7%) | 0.76 |
| | $25,000 to $49,999 | 6 (4.9%) | 1 (0.8%) | 11 (9.0%) | 45 (36.9%) | 59 (48.4%) | 122 (11.5%) | 0.64 |
| | $50,000 to $74,999 | 8 (5.0%) | 2 (1.3%) | 18 (11.3%) | 53 (33.3%) | 78 (49.1%) | 159 (15.0%) | 0.68 |
| | $75,000 to $99,999 | 3 (2.1%) | 9 (6.2%) | 25 (17.2%) | 58 (40.0%) | 50 (34.5%) | 145 (13.7%) | 0.18 |
| | Over $150,000 | 8 (4.2%) | 8 (4.2%) | 18 (9.5%) | 66 (34.9%) | 89 (47.1%) | 189 (17.8%) | 0.51 |
| | Prefer not to answer | 7 (5.4%) | 8 (6.2%) | 18 (13.8%) | 31 (23.8%) | 66 (50.8%) | 130 (12.3%) | 0.56 |
| Dietary Restriction | No dietary restriction | 31 (4.3%) | 30 (4.2%) | 88 (12.3%) | 253 (35.3%) | 315 (43.9%) | 717 (67.7%) | |
| | Others | 2 (4.9%) | 2 (4.9%) | 6 (14.6%) | 4 (9.8%) | 27 (65.9%) | 41 (3.9%) | 0.24 |
| | Yes, Allergies and/or intolerances, and faith-based restrictions (e.g. Halal, Kosher, etc.) | 2 (13.3%) | 2 (13.3%) | 3 (20.0%) | 5 (33.3%) | 3 (20.0%) | 15 (1.4%) | 0.02* |

| | | | | | | | | |
|---|---|---|---|---|---|---|---|---|
| | Yes, faith-based restrictions (e.g. Halal, Kosher, etc.) | 1 (5.9%) | 0 (0%) | 1 (5.9%) | 7 (41.2%) | 8 (47.1%) | 17 (1.6%) | 0.67 |
| | Yes, food allergies and/or intolerances | 14 (5.2%) | 11 (4.1%) | 25 (9.3%) | 81 (30.1%) | 138 (51.3%) | 269 (25.4%) | 0.36 |
| Eating out Frequency | Monthly | 15 (4.8%) | 12 (3.8%) | 24 (7.7%) | 99 (31.6%) | 163 (52.1%) | 313 (29.5%) | |
| | 2-3 times a week | 7 (4.7%) | 8 (5.4%) | 15 (10.1%) | 53 (35.8%) | 65 (43.9%) | 148 (14.0%) | 0.56 |
| | Bi-weekly | 15 (5.4%) | 10 (3.6%) | 38 (13.7%) | 92 (33.2%) | 122 (44.0%) | 277 (26.1%) | 0.18 |
| | Daily | 0 (0%) | 0 (0%) | 1 (5.0%) | 9 (45.0%) | 10 (50.0%) | 20 (1.9%) | 0.49 |
| | Weekly | 13 (4.3%) | 15 (5.0%) | 46 (15.2%) | 97 (32.1%) | 131 (43.4%) | 302 (28.5%) | 0.06 |
| | **Total** | **50 (4.7%)** | **45 (4.2%)** | **124 (11.7%)** | **350 (33.0%)** | **491 (46.3%)** | **1060 (100.0%)** | |
| **Consider the following statement: The presence of a health logo on menu influences my decisions to choose a restaurant.** | | | | | | | | |
| Age Group | The Silent Generation: Born 1928-1945 | 5 (20.0%) | 4 (16.0%) | 12 (48.0%) | 4 (16.0%) | 0 (0%) | 25 (2.4%) | |
| | Baby Boomers: Born 1946-1964 | 42 (12.5%) | 41 (12.2%) | 139 (41.2%) | 97 (28.8%) | 18 (5.3%) | 337 (32.7%) | 0.08 |
| | Generation X: Born 1965-1980 | 41 (17.7%) | 37 (16.0%) | 86 (37.2%) | 51 (22.1%) | 16 (6.9%) | 231 (22.4%) | 0.38 |
| | Generation Z: Born 1997-2012 | 8 (12.7%) | 10 (15.9%) | 29 (46.0%) | 12 (19.0%) | 4 (6.3%) | 63 (6.1%) | 0.18 |
| | Millennials: Born 1981-1996 | 79 (21.1%) | 62 (16.6%) | 133 (35.6%) | 91 (24.3%) | 9 (2.4%) | 374 (36.3%) | 0.70 |
| | Prefer not to answer | 0 (0%) | 0 (0%) | 1 (100.0%) | 0 (0%) | 0 (0%) | 1 (0.1%) | 0.89 |
| Gender | Male | 100 (19.9%) | 80 (15.9%) | 196 (39.0%) | 107 (21.3%) | 20 (4.0%) | 503 (48.8%) | |
| | Female | 74 (14.2%) | 74 (14.2%) | 200 (38.5%) | 146 (28.1%) | 26 (5.0%) | 520 (50.5%) | 0.01* |
| | Other | 0 (0%) | 0 (0%) | 2 (66.7%) | 1 (33.3%) | 0 (0%) | 3 (0.3%) | 0.26 |
| | Prefer not to answer | 1 (25.0%) | 0 (0%) | 1 (25.0%) | 1 (25.0%) | 1 (25.0%) | 4 (0.4%) | 0.33 |
| Marital Status | Single | 53 (19.2%) | 57 (20.7%) | 99 (35.9%) | 59 (21.4%) | 8 (2.9%) | 276 (26.8%) | |
| | Married | 96 (15.4%) | 86 (13.8%) | 252 (40.3%) | 159 (25.4%) | 32 (5.1%) | 625 (60.7%) | 0.02* |

| | | | | | | | | |
|---|---|---|---|---|---|---|---|---|
| | Separated / Divorced | 18 (23.1%) | 8 (10.3%) | 27 (34.6%) | 20 (25.6%) | 5 (6.4%) | 78 (7.6%) | 0.57 |
| | Widowed | 3 (13.0%) | 2 (8.7%) | 11 (47.8%) | 7 (30.4%) | 0 (0%) | 23 (2.2%) | 0.38 |
| | Prefer not to answer | 5 (18.5%) | 1 (3.7%) | 10 (37.0%) | 9 (33.3%) | 2 (7.4%) | 27 (2.6%) | 0.06 |
| Education | Did not graduate high school | 3 (20.0%) | 3 (20.0%) | 6 (40.0%) | 3 (20.0%) | 0 (0%) | 15 (1.5%) | |
| | Community college, technical college, or CEGEP | 35 (14.4%) | 30 (12.3%) | 103 (42.4%) | 63 (25.9%) | 12 (4.9%) | 243 (23.6%) | 0.25 |
| | High school graduate certificate or equivalent | 24 (18.3%) | 24 (18.3%) | 59 (45.0%) | 17 (13.0%) | 7 (5.3%) | 131 (12.7%) | 0.92 |
| | Post-graduate degree | 29 (16.3%) | 27 (15.2%) | 64 (36.0%) | 48 (27.0%) | 10 (5.6%) | 178 (17.3%) | 0.26 |
| | Trades certificate or diploma | 17 (20.2%) | 13 (15.5%) | 38 (45.2%) | 16 (19.0%) | 0 (0%) | 84 (8.2%) | 0.68 |
| | University (undergraduate degree) | 67 (17.7%) | 57 (15.0%) | 129 (34.0%) | 108 (28.5%) | 18 (4.7%) | 379 (36.8%) | 0.28 |
| Income | Under $25,000 | 7 (14.3%) | 9 (18.4%) | 19 (38.8%) | 11 (22.4%) | 3 (6.1%) | 49 (4.8%) | |
| | $100,000 to $124,000 | 24 (18.0%) | 20 (15.0%) | 56 (42.1%) | 30 (22.6%) | 3 (2.3%) | 133 (12.9%) | 0.31 |
| | $125,000 to $149,999 | 20 (16.4%) | 23 (18.9%) | 46 (37.7%) | 29 (23.8%) | 4 (3.3%) | 122 (11.8%) | 0.26 |
| | $25,000 to $49,999 | 24 (20.0%) | 16 (13.3%) | 43 (35.8%) | 31 (25.8%) | 6 (5.0%) | 120 (11.6%) | 0.62 |
| | $50,000 to $74,999 | 27 (17.9%) | 22 (14.6%) | 59 (39.1%) | 38 (25.2%) | 5 (3.3%) | 151 (14.6%) | 0.50 |
| | $75,000 to $99,999 | 22 (15.4%) | 19 (13.3%) | 66 (46.2%) | 30 (21.0%) | 6 (4.2%) | 143 (13.9%) | 0.52 |
| | Over $150,000 | 30 (16.1%) | 30 (16.1%) | 60 (32.3%) | 57 (30.6%) | 9 (4.8%) | 186 (18.0%) | 0.61 |
| | Prefer not to answer | 21 (16.5%) | 15 (11.8%) | 51 (40.2%) | 29 (22.8%) | 11 (8.7%) | 127 (12.3%) | 0.67 |
| Dietary Restriction | No dietary restriction | 128 (18.3%) | 109 (15.6%) | 269 (38.4%) | 165 (23.6%) | 29 (4.1%) | 700 (67.9%) | |
| | Others | 3 (8.1%) | 5 (13.5%) | 16 (43.2%) | 10 (27.0%) | 3 (8.1%) | 37 (3.6%) | 0.09 |
| | Yes, Allergies and/or intolerances, and | 4 (26.7%) | 2 (13.3%) | 2 (13.3%) | 7 (46.7%) | 0 (0%) | 15 (1.5%) | 0.69 |

| | | | | | | | | |
|---|---|---|---|---|---|---|---|---|
| | faith-based restrictions (e.g. Halal, Kosher, etc.) | | | | | | | |
| | Yes, faith-based restrictions (e.g. Halal, Kosher, etc.) | 3 (17.6%) | 0 (0%) | 6 (35.3%) | 6 (35.3%) | 2 (11.8%) | 17 (1.6%) | 0.07 |
| | Yes, food allergies and/or intolerances | 37 (14.1%) | 38 (14.5%) | 107 (40.8%) | 67 (25.6%) | 13 (5.0%) | 262 (25.4%) | 0.10 |
| Eating out Frequency | Monthly | 48 (15.8%) | 41 (13.5%) | 127 (41.9%) | 78 (25.7%) | 9 (3.0%) | 303 (29.4%) | |
| | 2-3 times a week | 22 (15.6%) | 25 (17.7%) | 54 (38.3%) | 34 (24.1%) | 6 (4.3%) | 141 (13.7%) | 0.54 |
| | Bi-weekly | 46 (17.2%) | 42 (15.7%) | 97 (36.2%) | 68 (25.4%) | 15 (5.6%) | 268 (26.0%) | 0.47 |
| | Daily | 3 (15.0%) | 5 (25.0%) | 7 (35.0%) | 4 (20.0%) | 1 (5.0%) | 20 (1.9%) | 0.90 |
| | Weekly | 56 (18.7%) | 41 (13.7%) | 115 (38.5%) | 71 (23.7%) | 16 (5.4%) | 299 (29.0%) | 0.53 |
| | **Total** | **175 (17.0%)** | **154 (14.9%)** | **400 (38.8%)** | **255 (24.7%)** | **47 (4.6%)** | **1031 (100.0%)** | |
| **Consider the following statement: The people I am eating with influence my decision to choose dishes with and without a health logo.** | | | | | | | | |
| Age Group | The Silent Generation: Born 1928-1945 | 7 (28.0%) | 3 (12.0%) | 11 (44.0%) | 4 (16.0%) | 0 (0%) | 25 (2.4%) | |
| | Baby Boomers: Born 1946-1964 | 133 (39.5%) | 57 (16.9%) | 103 (30.6%) | 39 (11.6%) | 5 (1.5%) | 337 (32.8%) | 0.26 |
| | Generation X: Born 1965-1980 | 81 (35.2%) | 45 (19.6%) | 64 (27.8%) | 34 (14.8%) | 6 (2.6%) | 230 (22.4%) | 0.26 |
| | Generation Z: Born 1997-2012 | 9 (14.5%) | 15 (24.2%) | 18 (29.0%) | 16 (25.8%) | 4 (6.5%) | 62 (6.0%) | 0.40 |
| | Millennials: Born 1981-1996 | 91 (24.3%) | 80 (21.4%) | 102 (27.3%) | 81 (21.7%) | 20 (5.3%) | 374 (36.3%) | 0.91 |
| | Prefer not to answer | 0 (0%) | 0 (0%) | 1 (100.0%) | 0 (0%) | 0 (0%) | 1 (0.1%) | 0.86 |
| Gender | Male | 150 (29.9%) | 101 (20.2%) | 144 (28.7%) | 87 (17.4%) | 19 (3.8%) | 501 (48.7%) | |
| | Female | 168 (32.3%) | 99 (19.0%) | 152 (29.2%) | 85 (16.3%) | 16 (3.1%) | 520 (50.6%) | 0.53 |

| | | | | | | | | |
|---|---|---|---|---|---|---|---|---|
| | Other | 1 (33.3%) | 0 (0%) | 1 (33.3%) | 1 (33.3%) | 0 (0%) | 3 (0.3%) | 0.44 |
| | Prefer not to answer | 2 (50.0%) | 0 (0%) | 1 (25.0%) | 1 (25.0%) | 0 (0%) | 4 (0.4%) | 0.80 |
| Marital Status | Single | 68 (24.6%) | 65 (23.6%) | 83 (30.1%) | 49 (17.8%) | 11 (4.0%) | 276 (26.9%) | |
| | Married | 203 (32.6%) | 118 (18.9%) | 174 (27.9%) | 108 (17.3%) | 20 (3.2%) | 623 (60.7%) | 0.90 |
| | Separated / Divorced | 36 (46.2%) | 12 (15.4%) | 20 (25.6%) | 8 (10.3%) | 2 (2.6%) | 78 (7.6%) | 0.27 |
| | Widowed | 7 (30.4%) | 3 (13.0%) | 9 (39.1%) | 4 (17.4%) | 0 (0%) | 23 (2.2%) | 0.50 |
| | Prefer not to answer | 6 (22.2%) | 2 (7.4%) | 12 (44.4%) | 5 (18.5%) | 2 (7.4%) | 27 (2.6%) | 0.05 |
| Education | Did not graduate high school | 7 (46.7%) | 2 (13.3%) | 3 (20.0%) | 2 (13.3%) | 1 (6.7%) | 15 (1.5%) | |
| | Community college, technical college, or CEGEP | 82 (33.7%) | 48 (19.8%) | 71 (29.2%) | 37 (15.2%) | 5 (2.1%) | 243 (23.6%) | 0.47 |
| | High school graduate certificate or equivalent | 52 (39.7%) | 19 (14.5%) | 45 (34.4%) | 12 (9.2%) | 3 (2.3%) | 131 (12.7%) | 0.75 |
| | Post-graduate degree | 59 (33.1%) | 34 (19.1%) | 46 (25.8%) | 33 (18.5%) | 6 (3.4%) | 178 (17.3%) | 0.48 |
| | Trades certificate or diploma | 24 (28.6%) | 17 (20.2%) | 34 (40.5%) | 7 (8.3%) | 2 (2.4%) | 84 (8.2%) | 0.53 |
| | University (undergraduate degree) | 97 (25.7%) | 80 (21.2%) | 99 (26.3%) | 83 (22.0%) | 18 (4.8%) | 377 (36.7%) | 0.22 |
| Income | Under $25,000 | 13 (26.5%) | 8 (16.3%) | 20 (40.8%) | 7 (14.3%) | 1 (2.0%) | 49 (4.8%) | |
| | $100,000 to $124,000 | 39 (29.3%) | 23 (17.3%) | 40 (30.1%) | 23 (17.3%) | 8 (6.0%) | 133 (12.9%) | 0.62 |
| | $125,000 to $149,999 | 43 (35.5%) | 20 (16.5%) | 32 (26.4%) | 21 (17.4%) | 5 (4.1%) | 121 (11.8%) | 0.22 |
| | $25,000 to $49,999 | 48 (40.0%) | 20 (16.7%) | 35 (29.2%) | 12 (10.0%) | 5 (4.2%) | 120 (11.7%) | 0.07 |
| | $50,000 to $74,999 | 45 (29.8%) | 35 (23.2%) | 46 (30.5%) | 21 (13.9%) | 4 (2.6%) | 151 (14.7%) | 0.35 |
| | $75,000 to $99,999 | 38 (26.6%) | 32 (22.4%) | 43 (30.1%) | 24 (16.8%) | 6 (4.2%) | 143 (13.9%) | 0.61 |
| | Over $150,000 | 53 (28.6%) | 37 (20.0%) | 42 (22.7%) | 50 (27.0%) | 3 (1.6%) | 185 (18.0%) | 0.87 |
| | Prefer not to answer | 42 (33.1%) | 25 (19.7%) | 41 (32.3%) | 16 (12.6%) | 3 (2.4%) | 127 (12.3%) | 0.26 |

| | | | | | | | | |
|---|---|---|---|---|---|---|---|---|
| Dietary Restriction | No dietary restriction | 221 (31.6%) | 131 (18.7%) | 206 (29.5%) | 119 (17.0%) | 22 (3.1%) | 699 (67.9%) | |
| | Others | 12 (32.4%) | 9 (24.3%) | 10 (27.0%) | 5 (13.5%) | 1 (2.7%) | 37 (3.6%) | 0.69 |
| | Yes, Allergies and/or intolerances, and faith-based restrictions (e.g. Halal, Kosher, etc.) | 4 (26.7%) | 5 (33.3%) | 1 (6.7%) | 3 (20.0%) | 2 (13.3%) | 15 (1.5%) | 0.81 |
| | Yes, faith-based restrictions (e.g. Halal, Kosher, et.) | 4 (23.5%) | 3 (17.6%) | 7 (41.2%) | 2 (11.8%) | 1 (5.9%) | 17 (1.7%) | 0.86 |
| | Yes, food allergies and/or intolerances | 80 (30.7%) | 52 (19.9%) | 75 (28.7%) | 45 (17.2%) | 9 (3.4%) | 261 (25.4%) | 0.90 |
| Eating out Frequency | Monthly | 108 (35.8%) | 61 (20.2%) | 89 (29.5%) | 42 (13.9%) | 2 (0.7%) | 302 (29.3%) | |
| | 2-3 times a week | 35 (24.8%) | 24 (17.0%) | 41 (29.1%) | 31 (22.0%) | 10 (7.1%) | 141 (13.7%) | 0.02* |
| | Bi-weekly | 79 (29.6%) | 62 (23.2%) | 72 (27.0%) | 45 (16.9%) | 9 (3.4%) | 267 (25.9%) | 0.36 |
| | Daily | 5 (25.0%) | 2 (10.0%) | 5 (25.0%) | 6 (30.0%) | 2 (10.0%) | 20 (1.9%) | 0.03* |
| | Weekly | 94 (31.4%) | 51 (17.1%) | 92 (30.8%) | 50 (16.7%) | 12 (4.0%) | 299 (29.1%) | 0.50 |
| | **Total** | **321 (31.2%)** | **200 (19.4%)** | **299 (29.1%)** | **174 (16.9%)** | **35 (3.4%)** | **1029 (100.0%)** | |
| **Consider the following statement: I would like to see a warning label like that for the prepackaged foods on restaurant menu items.** | | | | | | | | |
| Age Group | The Silent Generation: Born 1928-1945 | 1 (4.0%) | 1 (4.0%) | 10 (40.0%) | 8 (32.0%) | 5 (20.0%) | 25 (2.4%) | |
| | Baby Boomers: Born 1946-1964 | 17 (5.1%) | 26 (7.7%) | 72 (21.4%) | 123 (36.6%) | 98 (29.2%) | 336 (32.7%) | 0.33 |
| | Generation X: Born 1965-1980 | 23 (10.0%) | 19 (8.2%) | 62 (26.8%) | 73 (31.6%) | 54 (23.4%) | 231 (22.5%) | 0.85 |
| | Generation Z: Born 1997-2012 | 2 (3.2%) | 6 (9.5%) | 11 (17.5%) | 28 (44.4%) | 16 (25.4%) | 63 (6.1%) | 0.42 |

| | | | | | | | | |
|---|---|---|---|---|---|---|---|---|
| | Millennials: Born 1981-1996 | 36 (9.7%) | 36 (9.7%) | 94 (25.3%) | 131 (35.2%) | 75 (20.2%) | 372 (36.2%) | 0.72 |
| | Prefer not to answer | 0 (0%) | 0 (0%) | 0 (0%) | 0 (0%) | 1 (100.0%) | 1 (0.1%) | |
| Gender | Male | 41 (8.2%) | 41 (8.2%) | 148 (29.5%) | 162 (32.3%) | 109 (21.8%) | 501 (48.8%) | |
| | Female | 37 (7.1%) | 46 (8.9%) | 101 (19.5%) | 198 (38.2%) | 137 (26.4%) | 519 (50.5%) | 0.01* |
| | Other | 0 (0%) | 0 (0%) | 0 (0%) | 2 (66.7%) | 1 (33.3%) | 3 (0.3%) | 0.25 |
| | Prefer not to answer | 1 (25.0%) | 1 (25.0%) | 0 (0%) | 0 (0%) | 2 (50.0%) | 4 (0.4%) | 0.78 |
| Marital Status | Single | 19 (6.9%) | 25 (9.1%) | 72 (26.1%) | 96 (34.8%) | 64 (23.2%) | 276 (26.9%) | |
| | Married | 47 (7.6%) | 56 (9.0%) | 144 (23.2%) | 222 (35.7%) | 153 (24.6%) | 622 (60.6%) | 0.95 |
| | Separated / Divorced | 9 (11.5%) | 4 (5.1%) | 20 (25.6%) | 24 (30.8%) | 21 (26.9%) | 78 (7.6%) | 0.52 |
| | Widowed | 2 (8.7%) | 2 (8.7%) | 4 (17.4%) | 9 (39.1%) | 6 (26.1%) | 23 (2.2%) | 0.55 |
| | Prefer not to answer | 2 (7.4%) | 0 (0%) | 8 (29.6%) | 12 (44.4%) | 5 (18.5%) | 27 (2.6%) | 0.98 |
| Education | Did not graduate high school | 3 (20.0%) | 1 (6.7%) | 2 (13.3%) | 3 (20.0%) | 6 (40.0%) | 15 (1.5%) | |
| | Community college, technical college, or CEGEP | 20 (8.2%) | 15 (6.2%) | 68 (28.0%) | 87 (35.8%) | 53 (21.8%) | 243 (23.7%) | 0.78 |
| | High school graduate certificate or equivalent | 8 (6.2%) | 12 (9.3%) | 30 (23.3%) | 45 (34.9%) | 34 (26.4%) | 129 (12.6%) | 0.99 |
| | Post-graduate degree | 20 (11.3%) | 21 (11.9%) | 31 (17.5%) | 58 (32.8%) | 47 (26.6%) | 177 (17.2%) | 0.93 |
| | Trades certificate or diploma | 4 (4.8%) | 6 (7.1%) | 32 (38.1%) | 33 (39.3%) | 9 (10.7%) | 84 (8.2%) | 0.56 |
| | University (undergraduate degree) | 24 (6.3%) | 33 (8.7%) | 85 (22.4%) | 137 (36.1%) | 100 (26.4%) | 379 (36.9%) | 0.83 |
| Income | Under $25,000 | 3 (6.2%) | 4 (8.3%) | 16 (33.3%) | 10 (20.8%) | 15 (31.2%) | 48 (4.7%) | |
| | $100,000 to $124,000 | 11 (8.3%) | 12 (9.0%) | 31 (23.3%) | 48 (36.1%) | 31 (23.3%) | 133 (12.9%) | 0.92 |
| | $125,000 to $149,999 | 14 (11.5%) | 12 (9.8%) | 29 (23.8%) | 44 (36.1%) | 23 (18.9%) | 122 (11.9%) | 0.40 |
| | $25,000 to $49,999 | 12 (10.1%) | 7 (5.9%) | 21 (17.6%) | 45 (37.8%) | 34 (28.6%) | 119 (11.6%) | 0.60 |

| | | | | | | | | |
|---|---|---|---|---|---|---|---|---|
| | $50,000 to $74,999 | 11 (7.3%) | 15 (10.0%) | 40 (26.7%) | 45 (30.0%) | 39 (26.0%) | 150 (14.6%) | 0.81 |
| | $75,000 to $99,999 | 8 (5.6%) | 10 (6.9%) | 40 (27.8%) | 58 (40.3%) | 28 (19.4%) | 144 (14.0%) | 0.87 |
| | Over $150,000 | 10 (5.4%) | 19 (10.3%) | 44 (23.8%) | 65 (35.1%) | 47 (25.4%) | 185 (18.0%) | 0.79 |
| | Prefer not to answer | 10 (7.9%) | 9 (7.1%) | 28 (22.0%) | 48 (37.8%) | 32 (25.2%) | 127 (12.4%) | 0.81 |
| Dietary Restriction | No dietary restriction | 55 (7.9%) | 52 (7.4%) | 179 (25.6%) | 251 (36.0%) | 161 (23.1%) | 698 (68.0%) | |
| | Others | 3 (8.6%) | 2 (5.7%) | 7 (20.0%) | 9 (25.7%) | 14 (40.0%) | 35 (3.4%) | 0.23 |
| | Yes, Allergies and/or intolerances, and faith-based restrictions (e.g. Halal, Kosher, etc.) | 1 (6.7%) | 2 (13.3%) | 2 (13.3%) | 6 (40.0%) | 4 (26.7%) | 15 (1.5%) | 0.54 |
| | Yes, faith-based restrictions (e.g. Halal, Kosher, etc.) | 3 (17.6%) | 0 (0%) | 6 (35.3%) | 3 (17.6%) | 5 (29.4%) | 17 (1.7%) | 0.54 |
| | Yes, food allergies and/or intolerances | 17 (6.5%) | 31 (11.8%) | 55 (21.0%) | 94 (35.9%) | 65 (24.8%) | 262 (25.5%) | 0.84 |
| Eating out Frequency | Monthly | 24 (7.9%) | 20 (6.6%) | 62 (20.5%) | 115 (38.1%) | 81 (26.8%) | 302 (29.4%) | |
| | 2-3 times a week | 11 (7.7%) | 17 (12.0%) | 34 (23.9%) | 53 (37.3%) | 27 (19.0%) | 142 (13.8%) | 0.27 |
| | Bi-weekly | 15 (5.7%) | 24 (9.1%) | 81 (30.6%) | 81 (30.6%) | 64 (24.2%) | 265 (25.8%) | 0.40 |
| | Daily | 1 (5.0%) | 1 (5.0%) | 4 (20.0%) | 8 (40.0%) | 6 (30.0%) | 20 (1.9%) | 0.14 |
| | Weekly | 28 (9.4%) | 26 (8.7%) | 68 (22.7%) | 106 (35.5%) | 71 (23.7%) | 299 (29.1%) | 0.56 |
| | **Total** | **79 (7.7%)** | **88 (8.6%)** | **249 (24.2%)** | **363 (35.3%)** | **249 (24.2%)** | **1028 (100.0%)** | |

**If the warning labels on the menu for each of the nutrients of concern are designed as below (Fig 2), do you agree that it is more effective than the FOPL for packaged foods?**

| | | | | | | | | |
|---|---|---|---|---|---|---|---|---|
| Age Group | The Silent Generation: Born 1928-1945 | 2 (8.0%) | 0 (0%) | 6 (24.0%) | 5 (20.0%) | 12 (48.0%) | 25 (2.4%) | |
| | Baby Boomers: Born 1946-1964 | 17 (5.0%) | 17 (5.0%) | 79 (23.4%) | 127 (37.7%) | 97 (28.8%) | 337 (32.7%) | 0.09 |
| | Generation X: Born 1965-1980 | 12 (5.2%) | 16 (6.9%) | 61 (26.4%) | 88 (38.1%) | 54 (23.4%) | 231 (22.4%) | 0.02* |

| | | | | | | | | |
|---|---|---|---|---|---|---|---|---|
| | Generation Z: Born 1997-2012 | 2 (3.2%) | 4 (6.3%) | 16 (25.4%) | 19 (30.2%) | 22 (34.9%) | 63 (6.1%) | 0.24 |
| | Millennials: Born 1981-1996 | 24 (6.4%) | 41 (11.0%) | 70 (18.7%) | 149 (39.8%) | 90 (24.1%) | 374 (36.3%) | 0.02* |
| | Prefer not to answer | 0 (0%) | 0 (0%) | 1 (100.0%) | 0 (0%) | 0 (0%) | 1 (0.1%) | 0.18 |
| Gender | Male | 25 (5.0%) | 32 (6.4%) | 130 (25.8%) | 196 (39.0%) | 120 (23.9%) | 503 (48.8%) | |
| | Female | 31 (6.0%) | 45 (8.7%) | 101 (19.4%) | 190 (36.5%) | 153 (29.4%) | 520 (50.5%) | 0.14 |
| | Other | 0 (0%) | 0 (0%) | 1 (33.3%) | 1 (33.3%) | 1 (33.3%) | 3 (0.3%) | 0.60 |
| | Prefer not to answer | 1 (25.0%) | 1 (25.0%) | 1 (25.0%) | 0 (0%) | 1 (25.0%) | 4 (0.4%) | 0.12 |
| Marital Status | Single | 16 (5.8%) | 30 (10.9%) | 58 (21.0%) | 97 (35.1%) | 75 (27.2%) | 276 (26.8%) | |
| | Married | 31 (5.0%) | 39 (6.2%) | 149 (23.8%) | 242 (38.7%) | 164 (26.2%) | 625 (60.7%) | 0.68 |
| | Separated / Divorced | 6 (7.7%) | 4 (5.1%) | 15 (19.2%) | 28 (35.9%) | 25 (32.1%) | 78 (7.6%) | 0.46 |
| | Widowed | 1 (4.3%) | 1 (4.3%) | 6 (26.1%) | 9 (39.1%) | 6 (26.1%) | 23 (2.2%) | 0.92 |
| | Prefer not to answer | 2 (7.4%) | 4 (14.8%) | 5 (18.5%) | 12 (44.4%) | 4 (14.8%) | 27 (2.6%) | 0.37 |
| Education | Did not graduate high school | 1 (6.7%) | 2 (13.3%) | 3 (20.0%) | 3 (20.0%) | 6 (40.0%) | 15 (1.5%) | |
| | Community college, technical college, or CEGEP | 14 (5.8%) | 12 (4.9%) | 61 (25.1%) | 103 (42.4%) | 53 (21.8%) | 243 (23.6%) | 0.85 |
| | High school graduate certificate or equivalent | 9 (6.9%) | 9 (6.9%) | 41 (31.3%) | 32 (24.4%) | 40 (30.5%) | 131 (12.7%) | 0.84 |
| | Post-graduate degree | 11 (6.2%) | 21 (11.8%) | 28 (15.7%) | 65 (36.5%) | 53 (29.8%) | 178 (17.3%) | 0.85 |
| | Trades certificate or diploma | 6 (7.1%) | 6 (7.1%) | 24 (28.6%) | 34 (40.5%) | 14 (16.7%) | 84 (8.2%) | 0.49 |
| | University (undergraduate degree) | 16 (4.2%) | 28 (7.4%) | 76 (20.1%) | 150 (39.6%) | 109 (28.8%) | 379 (36.8%) | 0.73 |
| Income | Under $25,000 | 3 (6.1%) | 4 (8.2%) | 13 (26.5%) | 13 (26.5%) | 16 (32.7%) | 49 (4.8%) | |
| | $100,000 to $124,000 | 8 (6.0%) | 12 (9.0%) | 38 (28.6%) | 41 (30.8%) | 34 (25.6%) | 133 (12.9%) | 0.36 |

| | | | | | | | | |
|---|---|---|---|---|---|---|---|---|
| | $125,000 to $149,999 | 8 (6.6%) | 10 (8.2%) | 27 (22.1%) | 55 (45.1%) | 22 (18.0%) | 122 (11.8%) | 0.31 |
| | $25,000 to $49,999 | 11 (9.2%) | 5 (4.2%) | 25 (20.8%) | 41 (34.2%) | 38 (31.7%) | 120 (11.6%) | 0.95 |
| | $50,000 to $74,999 | 6 (4.0%) | 16 (10.6%) | 29 (19.2%) | 56 (37.1%) | 44 (29.1%) | 151 (14.6%) | 0.96 |
| | $75,000 to $99,999 | 4 (2.8%) | 7 (4.9%) | 40 (28.0%) | 55 (38.5%) | 37 (25.9%) | 143 (13.9%) | 1.00 |
| | Over $150,000 | 8 (4.3%) | 16 (8.6%) | 28 (15.1%) | 81 (43.5%) | 53 (28.5%) | 186 (18.0%) | 0.76 |
| | Prefer not to answer | 9 (7.1%) | 8 (6.3%) | 33 (26.0%) | 46 (36.2%) | 31 (24.4%) | 127 (12.3%) | 0.38 |
| Dietary Restriction | No dietary restriction | 33 (4.7%) | 46 (6.6%) | 162 (23.2%) | 278 (39.8%) | 180 (25.8%) | 699 (67.9%) | |
| | Others | 6 (16.2%) | 6 (16.2%) | 7 (18.9%) | 9 (24.3%) | 9 (24.3%) | 37 (3.6%) | 0.01* |
| | Yes, Allergies and/or intolerances, and faith-based restrictions (e.g. Halal, Kosher, etc.) | 1 (6.7%) | 3 (20.0%) | 1 (6.7%) | 6 (40.0%) | 4 (26.7%) | 15 (1.5%) | 0.64 |
| | Yes, faith-based restrictions (e.g. Halal, Kosher, etc.) | 0 (0%) | 0 (0%) | 2 (11.8%) | 9 (52.9%) | 6 (35.3%) | 17 (1.7%) | 0.13 |
| | Yes, food allergies and/or intolerances | 16 (6.1%) | 23 (8.8%) | 61 (23.3%) | 86 (32.8%) | 76 (29.0%) | 262 (25.4%) | 0.70 |
| Eating out Frequency | Monthly | 18 (5.9%) | 21 (6.9%) | 74 (24.4%) | 100 (33.0%) | 90 (29.7%) | 303 (29.4%) | |
| | 2-3 times a week | 6 (4.2%) | 10 (7.0%) | 31 (21.8%) | 60 (42.3%) | 35 (24.6%) | 142 (13.8%) | 0.47 |
| | Bi-weekly | 11 (4.1%) | 16 (6.0%) | 64 (24.0%) | 106 (39.7%) | 70 (26.2%) | 267 (25.9%) | 0.73 |
| | Daily | 2 (10.0%) | 2 (10.0%) | 3 (15.0%) | 7 (35.0%) | 6 (30.0%) | 20 (1.9%) | 0.52 |
| | Weekly | 20 (6.7%) | 29 (9.7%) | 61 (20.4%) | 115 (38.5%) | 74 (24.7%) | 299 (29.0%) | 0.63 |
| | **Total** | **57 (5.5%)** | **78 (7.6%)** | **233 (22.6%)** | **388 (37.6%)** | **275 (26.7%)** | **1031 (100.0%)** | |
| **Consider the following statement: I am more inclined to eat at a restaurant that has labels displayed on the menu.** | | | | | | | | |
| Age Group | The Silent Generation: Born 1928-1945 | 5 (20.0%) | 1 (4.0%) | 14 (56.0%) | 5 (20.0%) | 0 (0%) | 25 (2.4%) | |
| | Baby Boomers: Born 1946-1964 | 47 (13.9%) | 49 (14.5%) | 141 (41.8%) | 78 (23.1%) | 22 (6.5%) | 337 (32.7%) | 0.57 |

| | | | | | | | | |
|---|---|---|---|---|---|---|---|---|
| | Generation X: Born 1965-1980 | 38 (16.5%) | 27 (11.7%) | 104 (45.0%) | 48 (20.8%) | 14 (6.1%) | 231 (22.4%) | 0.93 |
| | Generation Z: Born 1997-2012 | 6 (9.5%) | 2 (3.2%) | 29 (46.0%) | 20 (31.7%) | 6 (9.5%) | 63 (6.1%) | 0.07 |
| | Millennials: Born 1981-1996 | 53 (14.1%) | 65 (17.3%) | 166 (44.3%) | 65 (17.3%) | 26 (6.9%) | 375 (36.3%) | 0.83 |
| | Prefer not to answer | 0 (0%) | 0 (0%) | 1 (100.0%) | 0 (0%) | 0 (0%) | 1 (0.1%) | 0.94 |
| Gender | Male | 83 (16.5%) | 67 (13.3%) | 228 (45.3%) | 98 (19.5%) | 27 (5.4%) | 503 (48.8%) | |
| | Female | 64 (12.3%) | 77 (14.8%) | 225 (43.2%) | 115 (22.1%) | 40 (7.7%) | 521 (50.5%) | 0.21 |
| | Other | 0 (0%) | 0 (0%) | 2 (66.7%) | 1 (33.3%) | 0 (0%) | 3 (0.3%) | 0.47 |
| | Prefer not to answer | 2 (50.0%) | 0 (0%) | 0 (0%) | 1 (25.0%) | 1 (25.0%) | 4 (0.4%) | 0.86 |
| Marital Status | Single | 39 (14.1%) | 38 (13.7%) | 125 (45.1%) | 56 (20.2%) | 19 (6.9%) | 277 (26.9%) | |
| | Married | 88 (14.1%) | 87 (13.9%) | 276 (44.2%) | 134 (21.4%) | 40 (6.4%) | 625 (60.7%) | 0.62 |
| | Separated / Divorced | 18 (23.1%) | 10 (12.8%) | 28 (35.9%) | 15 (19.2%) | 7 (9.0%) | 78 (7.6%) | 0.49 |
| | Widowed | 3 (13.0%) | 1 (4.3%) | 13 (56.5%) | 5 (21.7%) | 1 (4.3%) | 23 (2.2%) | 0.73 |
| | Prefer not to answer | 1 (3.7%) | 7 (25.9%) | 12 (44.4%) | 6 (22.2%) | 1 (3.7%) | 27 (2.6%) | 0.76 |
| Education | Did not graduate high school | 3 (20.0%) | 3 (20.0%) | 5 (33.3%) | 4 (26.7%) | 0 (0%) | 15 (1.5%) | |
| | Community college, technical college, or CEGEP | 44 (18.1%) | 25 (10.3%) | 104 (42.8%) | 57 (23.5%) | 13 (5.3%) | 243 (23.6%) | 0.37 |
| | High school graduate certificate or equivalent | 20 (15.3%) | 19 (14.5%) | 67 (51.1%) | 15 (11.5%) | 10 (7.6%) | 131 (12.7%) | 0.65 |
| | Post-graduate degree | 23 (12.9%) | 36 (20.2%) | 63 (35.4%) | 33 (18.5%) | 23 (12.9%) | 178 (17.3%) | 0.25 |
| | Trades certificate or diploma | 15 (17.9%) | 13 (15.5%) | 41 (48.8%) | 14 (16.7%) | 1 (1.2%) | 84 (8.1%) | 0.76 |
| | University (undergraduate degree) | 44 (11.6%) | 48 (12.6%) | 174 (45.8%) | 93 (24.5%) | 21 (5.5%) | 380 (36.9%) | 0.21 |

| | | | | | | | | |
|---|---|---|---|---|---|---|---|---|
| Income | Under $25,000 | 9 (18.4%) | 5 (10.2%) | 23 (46.9%) | 7 (14.3%) | 5 (10.2%) | 49 (4.7%) | |
| | $100,000 to $124,000 | 24 (18.0%) | 19 (14.3%) | 60 (45.1%) | 23 (17.3%) | 7 (5.3%) | 133 (12.9%) | 0.49 |
| | $125,000 to $149,999 | 21 (17.2%) | 19 (15.6%) | 50 (41.0%) | 25 (20.5%) | 7 (5.7%) | 122 (11.8%) | 0.68 |
| | $25,000 to $49,999 | 17 (14.2%) | 14 (11.7%) | 59 (49.2%) | 20 (16.7%) | 10 (8.3%) | 120 (11.6%) | 0.85 |
| | $50,000 to $74,999 | 19 (12.6%) | 26 (17.2%) | 65 (43.0%) | 34 (22.5%) | 7 (4.6%) | 151 (14.6%) | 1.00 |
| | $75,000 to $99,999 | 17 (11.8%) | 19 (13.2%) | 63 (43.8%) | 35 (24.3%) | 10 (6.9%) | 144 (14.0%) | 0.59 |
| | Over $150,000 | 27 (14.5%) | 28 (15.1%) | 70 (37.6%) | 46 (24.7%) | 15 (8.1%) | 186 (18.0%) | 0.67 |
| | Prefer not to answer | 15 (11.8%) | 14 (11.0%) | 65 (51.2%) | 26 (20.5%) | 7 (5.5%) | 127 (12.3%) | 0.91 |
| Dietary Restriction | No dietary restriction | 107 (15.3%) | 104 (14.9%) | 313 (44.7%) | 136 (19.4%) | 40 (5.7%) | 700 (67.9%) | |
| | Others | 4 (10.8%) | 8 (21.6%) | 15 (40.5%) | 5 (13.5%) | 5 (13.5%) | 37 (3.6%) | 0.80 |
| | Yes, Allergies and/or intolerances, and faith-based restrictions (e.g. Halal, Kosher, etc.) | 1 (6.7%) | 2 (13.3%) | 5 (33.3%) | 6 (40.0%) | 1 (6.7%) | 15 (1.5%) | 0.21 |
| | Yes, faith-based restrictions (e.g. Halal, Kosher, etc.) | 2 (11.8%) | 1 (5.9%) | 6 (35.3%) | 5 (29.4%) | 3 (17.6%) | 17 (1.6%) | 0.06 |
| | Yes, food allergies and/or intolerances | 35 (13.4%) | 29 (11.1%) | 116 (44.3%) | 63 (24.0%) | 19 (7.3%) | 262 (25.4%) | 0.05 |
| Eating out Frequency | Monthly | 43 (14.2%) | 39 (12.9%) | 141 (46.5%) | 62 (20.5%) | 18 (5.9%) | 303 (29.4%) | |
| | 2-3 times a week | 24 (16.9%) | 18 (12.7%) | 63 (44.4%) | 31 (21.8%) | 6 (4.2%) | 142 (13.8%) | 0.55 |
| | Bi-weekly | 44 (16.4%) | 45 (16.8%) | 108 (40.3%) | 51 (19.0%) | 20 (7.5%) | 268 (26.0%) | 0.42 |
| | Daily | 1 (5.0%) | 2 (10.0%) | 10 (50.0%) | 4 (20.0%) | 3 (15.0%) | 20 (1.9%) | 0.06 |
| | Weekly | 37 (12.4%) | 40 (13.4%) | 133 (44.5%) | 68 (22.7%) | 21 (7.0%) | 299 (29.0%) | 0.45 |
| | **Total** | **149 (14.4%)** | **144 (14.0%)** | **455 (44.1%)** | **216 (20.9%)** | **68 (6.6%)** | **1032 (100.0%)** | |

**Consider the following statement: I am willing to pay more for a meal at a restaurant to avoid menu items with warning labels.**

| | | | | | | | | |
|---|---|---|---|---|---|---|---|---|
| Age Group | The Silent Generation: Born 1928-1945 | 7 (28.0%) | 4 (16.0%) | 13 (52.0%) | 1 (4.0%) | 0 (0%) | 25 (2.4%) | |
| | Baby Boomers: Born 1946-1964 | 117 (34.8%) | 80 (23.8%) | 93 (27.7%) | 38 (11.3%) | 8 (2.4%) | 336 (32.6%) | 0.45 |
| | Generation X: Born 1965-1980 | 76 (32.9%) | 49 (21.2%) | 64 (27.7%) | 34 (14.7%) | 8 (3.5%) | 231 (22.4%) | 0.73 |
| | Generation Z: Born 1997-2012 | 15 (23.8%) | 20 (31.7%) | 16 (25.4%) | 10 (15.9%) | 2 (3.2%) | 63 (6.1%) | 0.81 |
| | Millennials: Born 1981-1996 | 117 (31.2%) | 89 (23.7%) | 107 (28.5%) | 53 (14.1%) | 9 (2.4%) | 375 (36.4%) | 0.68 |
| | Prefer not to answer | 1 (100.0%) | 0 (0%) | 0 (0%) | 0 (0%) | 0 (0%) | 1 (0.1%) | |
| Gender | Male | 168 (33.5%) | 126 (25.1%) | 140 (27.9%) | 61 (12.2%) | 7 (1.4%) | 502 (48.7%) | |
| | Female | 162 (31.1%) | 114 (21.9%) | 151 (29.0%) | 74 (14.2%) | 20 (3.8%) | 521 (50.6%) | 0.05 |
| | Other | 1 (33.3%) | 1 (33.3%) | 1 (33.3%) | 0 (0%) | 0 (0%) | 3 (0.3%) | 0.86 |
| | Prefer not to answer | 2 (50.0%) | 0 (0%) | 1 (25.0%) | 1 (25.0%) | 0 (0%) | 4 (0.4%) | 0.89 |
| Marital Status | Single | 89 (32.2%) | 75 (27.2%) | 74 (26.8%) | 30 (10.9%) | 8 (2.9%) | 276 (26.8%) | |
| | Married | 200 (32.0%) | 139 (22.2%) | 186 (29.8%) | 85 (13.6%) | 15 (2.4%) | 625 (60.7%) | 0.20 |
| | Separated / Divorced | 27 (34.6%) | 16 (20.5%) | 20 (25.6%) | 14 (17.9%) | 1 (1.3%) | 78 (7.6%) | 0.28 |
| | Widowed | 10 (43.5%) | 6 (26.1%) | 4 (17.4%) | 1 (4.3%) | 2 (8.7%) | 23 (2.2%) | 0.58 |
| | Prefer not to answer | 6 (22.2%) | 6 (22.2%) | 8 (29.6%) | 6 (22.2%) | 1 (3.7%) | 27 (2.6%) | 0.07 |
| Education | Did not graduate high school | 6 (40.0%) | 4 (26.7%) | 3 (20.0%) | 2 (13.3%) | 0 (0%) | 15 (1.5%) | |
| | Community college, technical college, or CEGEP | 80 (33.1%) | 59 (24.4%) | 71 (29.3%) | 24 (9.9%) | 8 (3.3%) | 242 (23.5%) | 0.54 |
| | High school graduate certificate or equivalent | 44 (33.6%) | 29 (22.1%) | 42 (32.1%) | 14 (10.7%) | 2 (1.5%) | 131 (12.7%) | 0.56 |
| | Post-graduate degree | 62 (34.8%) | 46 (25.8%) | 38 (21.3%) | 29 (16.3%) | 3 (1.7%) | 178 (17.3%) | 0.59 |

| | | | | | | | | |
|---|---|---|---|---|---|---|---|---|
| | Trades certificate or diploma | 30 (35.7%) | 17 (20.2%) | 28 (33.3%) | 8 (9.5%) | 1 (1.2%) | 84 (8.2%) | 0.60 |
| | University (undergraduate degree) | 111 (29.2%) | 87 (22.9%) | 110 (28.9%) | 59 (15.5%) | 13 (3.4%) | 380 (36.9%) | 0.31 |
| Income | Under $25,000 | 19 (39.6%) | 10 (20.8%) | 12 (25.0%) | 7 (14.6%) | 0 (0%) | 48 (4.7%) | |
| | $100,000 to $124,000 | 46 (34.6%) | 30 (22.6%) | 32 (24.1%) | 20 (15.0%) | 5 (3.8%) | 133 (12.9%) | 0.58 |
| | $125,000 to $149,999 | 44 (36.1%) | 26 (21.3%) | 34 (27.9%) | 13 (10.7%) | 5 (4.1%) | 122 (11.8%) | 0.77 |
| | $25,000 to $49,999 | 43 (35.8%) | 23 (19.2%) | 37 (30.8%) | 16 (13.3%) | 1 (0.8%) | 120 (11.6%) | 0.58 |
| | $50,000 to $74,999 | 51 (33.8%) | 39 (25.8%) | 40 (26.5%) | 19 (12.6%) | 2 (1.3%) | 151 (14.6%) | 0.57 |
| | $75,000 to $99,999 | 36 (25.0%) | 46 (31.9%) | 37 (25.7%) | 21 (14.6%) | 4 (2.8%) | 144 (14.0%) | 0.22 |
| | Over $150,000 | 56 (30.1%) | 42 (22.6%) | 56 (30.1%) | 24 (12.9%) | 8 (4.3%) | 186 (18.0%) | 0.33 |
| | Prefer not to answer | 38 (29.9%) | 26 (20.5%) | 45 (35.4%) | 16 (12.6%) | 2 (1.6%) | 127 (12.3%) | 0.28 |
| Dietary Restriction | No dietary restriction | 228 (32.6%) | 176 (25.2%) | 189 (27.0%) | 89 (12.7%) | 17 (2.4%) | 699 (67.9%) | |
| | Others | 10 (27.0%) | 7 (18.9%) | 13 (35.1%) | 6 (16.2%) | 1 (2.7%) | 37 (3.6%) | 0.23 |
| | Yes, Allergies and/or intolerances, and faith-based restrictions (e.g. Halal, Kosher, etc.) | 2 (13.3%) | 4 (26.7%) | 5 (33.3%) | 2 (13.3%) | 2 (13.3%) | 15 (1.5%) | 0.10 |
| | Yes, faith-based restrictions (e.g. Halal, Kosher, etc.) | 3 (17.6%) | 4 (23.5%) | 9 (52.9%) | 1 (5.9%) | 0 (0%) | 17 (1.7%) | 0.69 |
| | Yes, food allergies and/or intolerances | 90 (34.4%) | 51 (19.5%) | 76 (29.0%) | 38 (14.5%) | 7 (2.7%) | 262 (25.4%) | 0.93 |
| Eating out Frequency | Monthly | 106 (35.1%) | 68 (22.5%) | 89 (29.5%) | 34 (11.3%) | 5 (1.7%) | 302 (29.3%) | |
| | 2-3 times a week | 40 (28.2%) | 33 (23.2%) | 45 (31.7%) | 20 (14.1%) | 4 (2.8%) | 142 (13.8%) | 0.21 |
| | Bi-weekly | 87 (32.5%) | 65 (24.3%) | 78 (29.1%) | 29 (10.8%) | 9 (3.4%) | 268 (26.0%) | 0.53 |
| | Daily | 5 (25.0%) | 3 (15.0%) | 5 (25.0%) | 5 (25.0%) | 2 (10.0%) | 20 (1.9%) | 0.02* |

| | | | | | | | | |
|---|---|---|---|---|---|---|---|---|
| | Weekly | 95 (31.8%) | 73 (24.4%) | 76 (25.4%) | 48 (16.1%) | 7 (2.3%) | 299 (29.0%) | 0.48 |
| | **Total** | **333 (32.3%)** | **242 (23.5%)** | **293 (28.4%)** | **136 (13.2%)** | **27 (2.6%)** | **1031 (100.0%)** | |
| **Consider the following statement: I think using warning labels on menu items will be effective informing consumers of the nutrition profile of a food.** | | | | | | | | |
| Age Group | The Silent Generation: Born 1928-1945 | 2 (8.0%) | 2 (8.0%) | 8 (32.0%) | 9 (36.0%) | 4 (16.0%) | 25 (2.4%) | |
| | Baby Boomers: Born 1946-1964 | 21 (6.3%) | 38 (11.3%) | 53 (15.8%) | 160 (47.8%) | 63 (18.8%) | 335 (32.7%) | 0.27 |
| | Generation X: Born 1965-1980 | 22 (9.6%) | 26 (11.4%) | 41 (18.0%) | 101 (44.3%) | 38 (16.7%) | 228 (22.3%) | 0.94 |
| | Generation Z: Born 1997-2012 | 1 (1.6%) | 5 (7.9%) | 8 (12.7%) | 35 (55.6%) | 14 (22.2%) | 63 (6.2%) | 0.19 |
| | Millennials: Born 1981-1996 | 22 (5.9%) | 44 (11.8%) | 76 (20.4%) | 176 (47.3%) | 54 (14.5%) | 372 (36.3%) | 0.94 |
| | Prefer not to answer | 0 (0%) | 0 (0%) | 0 (0%) | 1 (100.0%) | 0 (0%) | 1 (0.1%) | 0.57 |
| Gender | Male | 39 (7.8%) | 57 (11.4%) | 103 (20.7%) | 230 (46.2%) | 69 (13.9%) | 498 (48.6%) | |
| | Female | 28 (5.4%) | 58 (11.2%) | 83 (16.0%) | 247 (47.6%) | 103 (19.8%) | 519 (50.7%) | 0.003** |
| | Other | 0 (0%) | 0 (0%) | 0 (0%) | 2 (66.7%) | 1 (33.3%) | 3 (0.3%) | 0.07 |
| | Prefer not to answer | 1 (25.0%) | 0 (0%) | 0 (0%) | 3 (75.0%) | 0 (0%) | 4 (0.4%) | 0.89 |
| Marital Status | Single | 18 (6.6%) | 26 (9.5%) | 49 (17.9%) | 132 (48.4%) | 48 (17.6%) | 273 (26.7%) | |
| | Married | 39 (6.3%) | 75 (12.0%) | 115 (18.5%) | 289 (46.4%) | 105 (16.9%) | 623 (61.0%) | 0.75 |
| | Separated / Divorced | 6 (7.9%) | 8 (10.5%) | 13 (17.1%) | 34 (44.7%) | 15 (19.7%) | 76 (7.4%) | 0.75 |
| | Widowed | 3 (13.0%) | 2 (8.7%) | 3 (13.0%) | 12 (52.2%) | 3 (13.0%) | 23 (2.3%) | 0.36 |
| | Prefer not to answer | 2 (7.4%) | 3 (11.1%) | 6 (22.2%) | 14 (51.9%) | 2 (7.4%) | 27 (2.6%) | 0.21 |
| Education | Did not graduate high school | 1 (6.7%) | 3 (20.0%) | 3 (20.0%) | 6 (40.0%) | 2 (13.3%) | 15 (1.5%) | |
| | Community college, technical college, or CEGEP | 19 (7.9%) | 28 (11.7%) | 45 (18.8%) | 107 (44.6%) | 41 (17.1%) | 240 (23.5%) | 0.48 |

| | | | | | | | | |
|---|---|---|---|---|---|---|---|---|
| | High school graduate certificate or equivalent | 14 (10.7%) | 9 (6.9%) | 25 (19.1%) | 59 (45.0%) | 24 (18.3%) | 131 (12.8%) | 0.43 |
| | Post-graduate degree | 12 (6.7%) | 32 (18.0%) | 25 (14.0%) | 79 (44.4%) | 30 (16.9%) | 178 (17.4%) | 0.49 |
| | Trades certificate or diploma | 7 (8.5%) | 8 (9.8%) | 21 (25.6%) | 38 (46.3%) | 8 (9.8%) | 82 (8.0%) | 0.85 |
| | University (undergraduate degree) | 15 (4.0%) | 35 (9.3%) | 66 (17.5%) | 193 (51.2%) | 68 (18.0%) | 377 (36.9%) | 0.19 |
| Income | Under $25,000 | 5 (10.4%) | 3 (6.2%) | 9 (18.8%) | 18 (37.5%) | 13 (27.1%) | 48 (4.7%) | |
| | $100,000 to $124,000 | 12 (9.0%) | 11 (8.3%) | 21 (15.8%) | 67 (50.4%) | 22 (16.5%) | 133 (13.0%) | 0.48 |
| | $125,000 to $149,999 | 8 (6.6%) | 15 (12.3%) | 23 (18.9%) | 55 (45.1%) | 21 (17.2%) | 122 (11.9%) | 0.41 |
| | $25,000 to $49,999 | 8 (6.7%) | 9 (7.6%) | 22 (18.5%) | 58 (48.7%) | 22 (18.5%) | 119 (11.6%) | 0.86 |
| | $50,000 to $74,999 | 9 (6.0%) | 20 (13.4%) | 27 (18.1%) | 67 (45.0%) | 26 (17.4%) | 149 (14.6%) | 0.40 |
| | $75,000 to $99,999 | 8 (5.7%) | 22 (15.6%) | 30 (21.3%) | 64 (45.4%) | 17 (12.1%) | 141 (13.8%) | 0.09 |
| | Over $150,000 | 11 (5.9%) | 25 (13.5%) | 27 (14.6%) | 87 (47.0%) | 35 (18.9%) | 185 (18.1%) | 0.67 |
| | Prefer not to answer | 7 (5.5%) | 10 (7.9%) | 27 (21.3%) | 66 (52.0%) | 17 (13.4%) | 127 (12.4%) | 0.29 |
| Dietary Restriction | No dietary restriction | 47 (6.8%) | 73 (10.5%) | 129 (18.6%) | 336 (48.4%) | 109 (15.7%) | 694 (67.8%) | |
| | Others | 3 (8.1%) | 8 (21.6%) | 3 (8.1%) | 16 (43.2%) | 7 (18.9%) | 37 (3.6%) | 0.58 |
| | Yes, Allergies and/or intolerances, and faith-based restrictions (e.g. Halal, Kosher, etc.) | 0 (0%) | 0 (0%) | 4 (26.7%) | 7 (46.7%) | 4 (26.7%) | 15 (1.5%) | 0.28 |
| | Yes, faith-based restrictions (e.g. Halal, Kosher, etc.) | 2 (11.8%) | 2 (11.8%) | 3 (17.6%) | 7 (41.2%) | 3 (17.6%) | 17 (1.7%) | 0.41 |
| | Yes, food allergies and/or intolerances | 16 (6.1%) | 32 (12.3%) | 47 (18.0%) | 116 (44.4%) | 50 (19.2%) | 261 (25.5%) | 0.98 |
| | Monthly | 27 (8.9%) | 33 (10.9%) | 51 (16.8%) | 152 (50.2%) | 40 (13.2%) | 303 (29.6%) | |

| | | | | | | | | |
|---|---|---|---|---|---|---|---|---|
| Eating out Frequency | 2-3 times a week | 6 (4.3%) | 17 (12.1%) | 23 (16.4%) | 69 (49.3%) | 25 (17.9%) | 140 (13.7%) | 0.09 |
| | Bi-weekly | 19 (7.2%) | 35 (13.3%) | 58 (22.1%) | 108 (41.1%) | 43 (16.3%) | 263 (25.7%) | 0.84 |
| | Daily | 1 (5.0%) | 1 (5.0%) | 5 (25.0%) | 9 (45.0%) | 4 (20.0%) | 20 (2.0%) | 0.19 |
| | Weekly | 15 (5.0%) | 29 (9.7%) | 49 (16.4%) | 144 (48.3%) | 61 (20.5%) | 298 (29.1%) | 0.006** |
| | **Total** | **68 (6.6%)** | **115 (11.2%)** | **186 (18.2%)** | **482 (47.1%)** | **173 (16.9%)** | **1024 (100.0%)** | |
| **Consider the following statement: If I see a warning label on a menu item, I will still consider buying it/going to that restaurant.** | | | | | | | | |
| Age Group | The Silent Generation: Born 1928-1945 | 0 (0%) | 4 (16.0%) | 9 (36.0%) | 8 (32.0%) | 4 (16.0%) | 25 (2.4%) | |
| | Baby Boomers: Born 1946-1964 | 11 (3.3%) | 37 (11.0%) | 90 (26.8%) | 152 (45.2%) | 46 (13.7%) | 336 (32.6%) | 0.92 |
| | Generation X: Born 1965-1980 | 5 (2.2%) | 15 (6.5%) | 66 (28.6%) | 99 (42.9%) | 46 (19.9%) | 231 (22.4%) | 0.49 |
| | Generation Z: Born 1997-2012 | 1 (1.6%) | 6 (9.5%) | 17 (27.0%) | 25 (39.7%) | 14 (22.2%) | 63 (6.1%) | 0.40 |
| | Millennials: Born 1981-1996 | 11 (2.9%) | 27 (7.2%) | 96 (25.7%) | 159 (42.5%) | 81 (21.7%) | 374 (36.3%) | 0.42 |
| | Prefer not to answer | 0 (0%) | 0 (0%) | 0 (0%) | 1 (100.0%) | 0 (0%) | 1 (0.1%) | 0.35 |
| Gender | Male | 15 (3.0%) | 36 (7.2%) | 148 (29.5%) | 209 (41.7%) | 93 (18.6%) | 501 (48.7%) | |
| | Female | 12 (2.3%) | 53 (10.2%) | 127 (24.4%) | 233 (44.7%) | 96 (18.4%) | 521 (50.6%) | 0.44 |
| | Other | 1 (33.3%) | 0 (0%) | 2 (66.7%) | 0 (0%) | 0 (0%) | 3 (0.3%) | 0.05 |
| | Prefer not to answer | 0 (0%) | 0 (0%) | 0 (0%) | 2 (50.0%) | 2 (50.0%) | 4 (0.4%) | 0.08 |
| Marital Status | Single | 9 (3.3%) | 24 (8.7%) | 75 (27.2%) | 109 (39.5%) | 59 (21.4%) | 276 (26.8%) | |
| | Married | 13 (2.1%) | 52 (8.3%) | 161 (25.8%) | 287 (46.0%) | 111 (17.8%) | 624 (60.7%) | 0.51 |
| | Separated / Divorced | 3 (3.8%) | 7 (9.0%) | 24 (30.8%) | 27 (34.6%) | 17 (21.8%) | 78 (7.6%) | 0.78 |
| | Widowed | 1 (4.3%) | 3 (13.0%) | 9 (39.1%) | 9 (39.1%) | 1 (4.3%) | 23 (2.2%) | 0.17 |
| | Prefer not to answer | 2 (7.4%) | 3 (11.1%) | 9 (33.3%) | 11 (40.7%) | 2 (7.4%) | 27 (2.6%) | 0.10 |
| Education | Did not graduate high school | 0 (0%) | 6 (40.0%) | 4 (26.7%) | 3 (20.0%) | 2 (13.3%) | 15 (1.5%) | |

| | | | | | | | | |
|---|---|---|---|---|---|---|---|---|
| | Community college, technical college, or CEGEP | 4 (1.6%) | 23 (9.5%) | 70 (28.8%) | 102 (42.0%) | 44 (18.1%) | 243 (23.6%) | 0.02* |
| | High school graduate certificate or equivalent | 4 (3.1%) | 10 (7.6%) | 38 (29.0%) | 54 (41.2%) | 25 (19.1%) | 131 (12.7%) | 0.02* |
| | Post-graduate degree | 7 (4.0%) | 15 (8.5%) | 40 (22.6%) | 78 (44.1%) | 37 (20.9%) | 177 (17.2%) | 0.02* |
| | Trades certificate or diploma | 3 (3.6%) | 4 (4.8%) | 27 (32.1%) | 36 (42.9%) | 14 (16.7%) | 84 (8.2%) | 0.04* |
| | University (undergraduate degree) | 10 (2.6%) | 31 (8.2%) | 98 (25.9%) | 171 (45.1%) | 69 (18.2%) | 379 (36.8%) | 0.02* |
| Income | Under $25,000 | 0 (0%) | 5 (10.2%) | 14 (28.6%) | 22 (44.9%) | 8 (16.3%) | 49 (4.8%) | |
| | $100,000 to $124,000 | 5 (3.8%) | 6 (4.5%) | 40 (30.1%) | 49 (36.8%) | 33 (24.8%) | 133 (12.9%) | 0.76 |
| | $125,000 to $149,999 | 2 (1.7%) | 4 (3.3%) | 36 (29.8%) | 52 (43.0%) | 27 (22.3%) | 121 (11.7%) | 0.70 |
| | $25,000 to $49,999 | 4 (3.3%) | 12 (10.0%) | 29 (24.2%) | 55 (45.8%) | 20 (16.7%) | 120 (11.7%) | 0.95 |
| | $50,000 to $74,999 | 7 (4.6%) | 18 (11.9%) | 32 (21.2%) | 67 (44.4%) | 27 (17.9%) | 151 (14.7%) | 0.95 |
| | $75,000 to $99,999 | 6 (4.2%) | 18 (12.6%) | 34 (23.8%) | 60 (42.0%) | 25 (17.5%) | 143 (13.9%) | 0.56 |
| | Over $150,000 | 1 (0.5%) | 17 (9.1%) | 48 (25.8%) | 84 (45.2%) | 36 (19.4%) | 186 (18.1%) | 0.87 |
| | Prefer not to answer | 3 (2.4%) | 9 (7.1%) | 45 (35.4%) | 55 (43.3%) | 15 (11.8%) | 127 (12.3%) | 0.39 |
| Dietary Restriction | No dietary restriction | 18 (2.6%) | 55 (7.9%) | 195 (27.9%) | 306 (43.8%) | 125 (17.9%) | 699 (67.9%) | |
| | Others | 0 (0%) | 1 (2.7%) | 11 (29.7%) | 19 (51.4%) | 6 (16.2%) | 37 (3.6%) | 0.47 |
| | Yes, Allergies and/or intolerances, and faith-based restrictions (e.g. Halal, Kosher, etc.) | 1 (6.7%) | 1 (6.7%) | 6 (40.0%) | 5 (33.3%) | 2 (13.3%) | 15 (1.5%) | 0.27 |
| | Yes, faith-based restrictions (e.g. Halal, Kosher, etc.) | 2 (11.8%) | 1 (5.9%) | 4 (23.5%) | 7 (41.2%) | 3 (17.6%) | 17 (1.7%) | 0.37 |

| | | | | | | | | |
|---|---|---|---|---|---|---|---|---|
| | Yes, food allergies and/or intolerances | 6 (2.3%) | 31 (11.9%) | 62 (23.8%) | 107 (41.0%) | 55 (21.1%) | 261 (25.4%) | 0.97 |
| Eating out Frequency | Monthly | 9 (3.0%) | 21 (6.9%) | 93 (30.7%) | 137 (45.2%) | 43 (14.2%) | 303 (29.4%) | |
| | 2-3 times a week | 3 (2.1%) | 13 (9.2%) | 33 (23.2%) | 56 (39.4%) | 37 (26.1%) | 142 (13.8%) | 0.13 |
| | Bi-weekly | 8 (3.0%) | 27 (10.1%) | 67 (25.0%) | 116 (43.3%) | 50 (18.7%) | 268 (26.0%) | 0.88 |
| | Daily | 1 (5.3%) | 1 (5.3%) | 3 (15.8%) | 9 (47.4%) | 5 (26.3%) | 19 (1.8%) | 0.33 |
| | Weekly | 7 (2.3%) | 27 (9.1%) | 82 (27.5%) | 126 (42.3%) | 56 (18.8%) | 298 (28.9%) | 0.96 |
| | **Total** | **28 (2.7%)** | **89 (8.6%)** | **277 (26.9%)** | **444 (43.1%)** | **191 (18.6%)** | **1029 (100.0%)** | |

*Note: Frequency and percentage were reported. Logistic ordinal regression was used to assess the difference with the reference level (first level in each group). *p<0.05, ** p<0.1, ***p<0.0001.*
